# The triad of knowledge, regular medical consultation, and recommendations for enhanced breast cancer screening intention in Morocco

**DOI:** 10.1007/s00432-024-05609-5

**Published:** 2024-01-31

**Authors:** Nadia Ouzennou, Mohamed Aboufaras, Nezha Nacer, Hicham Mejdouli, Jaouad Chouikh, Samia Rkha

**Affiliations:** 1 Institut Supérieur des Professions Infirmières et Techniques de Santé (ISPITS), Marrakech, Morocco; 2 Institut Supérieur des Professions Infirmières et Techniques de Santé (ISPITS), Higher Institute of Nursing and Technical Health Professions (ISPITS), Beni Mellal, Morocco; 3 Institut Supérieur des Professions Infirmières et Techniques de Santé (ISPITS), Essaouira, Morocco; 4 Laboratoire de Pharmacologie, Neurobiologie, Anthropobiologie, Environnement et Comportements, Faculté des Sciences Semlalia, Université Cadi Ayyad, Marrakech, Morocco

**Keywords:** Breast cancer, Intention, Knowledge, Screening

## Abstract

**Purpose:**

The low rate of breast cancer screening in Morocco is linked to delayed diagnosis and increased mortality rates. Furthermore, there is a lack of research on the association between screening intention and knowledge levels. Our objective was to assess the level of knowledge regarding breast cancer and screening, identify the determinants of this knowledge, and determine predictive factors for screening intention.

**Methods:**

A cross-sectional analytical study was conducted on 1014 women in the city of Essaouira, Morocco, during the years 2018–2020. A stepwise logistic regression analysis was performed to identify the predictors using SPSS software.

**Results:**

We found an above-average level of knowledge (67%). These knowledge levels were associated with several personal characteristics. Moreover, these knowledge levels proved to be powerful predictive factors for the intention to undergo screening, along with regular medical consultations and recommendations to undergo screening.

**Conclusion:**

Women had a high level of knowledge about breast cancer and its characteristics. These knowledge levels were strongly associated with the intention to undergo screening. However, despite this, the low participation rate encourages the reinforcement of research to align knowledge, intention, and screening practices.

## Introduction

In Morocco, according to the cancer registry of the Grand Casablanca Region for the year 2004 (Ministère de la Santé et Association Lalla Salma de Lutte contre le Cancer 2020), breast cancer is the leading cancer in women, accounting for 36.1% of all female cancers. A recent study in the Marrakech region has shown that this cancer represents over 40% of cancers in women and 25% in both sexes combined (Belbaraka et al. 2022). At an advanced stage, this disease can be fatal. However, early detection allows for a cure or at least a better prognosis. In this regard, the Ministry of Health in Morocco, in collaboration with the Lalla Salma Association for the Fight against Cancer, has implemented a National Cancer Prevention and Control Plan (2020–2029), following the global strategy. Its main objectives, among others, are to reduce cancer-related morbidity and mortality, improve the quality of life of patients, and combat risk factors (Ministère de la santé Marocain and Fondation Lalla Salma de prévention et du traitement contre le cancer, 2020).

The early detection activities are very important in reducing cancer-related mortality and represent a priority axis for the Moroccan Ministry of Health (Ministère de la santé Marocain and Fondation Lalla Salma de prévention et du traitement contre le cancer 2020). Early detection activities can reduce the burden of cancer and treatment by about one-third if the disease is detected in its early stages, where treatment is most effective. However, this can only be achieved if the population has a satisfactory level of knowledge and information about the disease, its clinical signs, and the possibilities of early detection. Unfortunately, in the Arab world and in Morocco, the level of participation in early detection activities and the level of knowledge among women regarding breast cancer remain insufficient to meet the challenge of the disease (Azaiza and Cohen 2006; Fakir et al. 2015). In Morocco, the participation rate in breast cancer screening was 35.7% (El Fakir et al. 2015). The situation becomes even more complicated in areas of social vulnerability, such as our study area (the province of Essaouira), where illiteracy affects almost half of the population, with 60.1% of women being illiterate (Haut commissariat au plan 2014). Indeed, in such conditions, the level of knowledge among women about breast cancer becomes a barrier to their participation in awareness and early detection campaigns. As a result, cancer is often diagnosed very late in Africa in general, and in Morocco, in particular (Espina et al. 2017).

Since these knowledge levels can influence the participation rate in breast cancer screening, and as no study, to our knowledge, has evaluated the intention to undergo breast cancer screening in Morocco and its predictors, our objective for this study was to assess the level of knowledge among women regarding breast cancer and its factors, as well as to identify predictive factors for the intention to undergo breast cancer screening among women in the province of Essaouira. Therefore, this research aims to address the following seven questions:

(A) What specific areas of breast cancer do women in the province of Essaouira tend to know better or less well?
(B) How does knowledge of breast cancer risk factors vary among different demographic categories of women in the province?
(C) What are the main channels of information used by women to acquire knowledge about breast cancer in the province?
(D) What is the perception of women in the province of Essaouira regarding the effectiveness of breast cancer screening for early detection?
(E) How do socio-economic determinants influence the level of knowledge among women regarding breast cancer and screening in the region?
(F) What are the main perceived barriers by women in the province of Essaouira regarding their intention to undergo breast cancer screening?
(G) Are there significant differences in the intention to undergo screening among women based on their sociodemographic, health, economic, and cognitive characteristics?

## Subjects and methods

### Design and participants

This is a descriptive and analytical cross-sectional survey conducted through structured interviews with adult women residing in the city of Essaouira, Morocco. The study was carried out from 2018 until the year 2020. The sample size was calculated based on the target population (*N* = 39,394) with a confidence level of 99%, using the following method:

$${\text{Sample}}\;{\text{size}}\left( n \right) = \frac{{z^{2} \; \times p\left( {1 - p} \right)/e^{2} }}{{1 + \left( {\frac{{z^{2} \times p\left( {1 - p} \right)}}{{e^{2} N}}} \right)}},$$

*n* is the sample size, *N* is the population size =39 394, *z* is the *z*-score =2.58, *e* is the error margin = 5%, *p* is the standard deviation = 0.5

Therefore, we have found a minimum sample size of 655.

### Data collection and ethical considerations

The structured interview allowed us to have direct contact with the women, making it easier for them to understand the questions and ensuring the credibility of their responses. Additionally, we chose a large sample size of women to gather maximum information and generalize the results among Souiri women (*n* = 1014).

The sampling method employed across the entire city, encompassing all its administrative districts, was a convenience sampling. Convenience sampling, a non-random selection, was chosen due to practical considerations related to accessibility and cost. Importantly, the absence of a pre-established list of the target population compelled us to opt for a non-random sampling approach. Thus, the recruitment of women was intentionally conducted at the broader level of the city and its administrative subdivisions.

All required ethical considerations were respected. The women were informed about the objective and process of the survey. The survey was conducted with utmost respect for the confidentiality of information and privacy.

The gathered information includes demographic, socio-economic, and cultural characteristics of the women, as well as their knowledge about breast cancer, risk factors, causes, detection methods, and their behaviors concerning self-examination, mammography, regular check-ups, and adherence to free early detection programs.

### Data analysis

To assess women's knowledge about breast cancer, 60% of correct responses on breast cancer, its risk factors, symptoms, prevention, treatment, self-examination, diagnosis, the availability of public health screening, and the recommended age for screening were considered as "good knowledge," while 40% or less of correct responses were considered as "poor knowledge."

Intentions for undergoing breast cancer screening were evaluated through the question: "Do you intend to undergo regular breast cancer screening?" Women who answered "Yes" were categorized as having "intention," while those who responded "No" were classified as "no intention."

Statistical analyses were conducted using SPSS software. A descriptive analysis was performed to describe the participants' characteristics and the rates of women's knowledge. Subsequently, a bivariate analysis using the Chi-square test was conducted to identify determinants of breast cancer knowledge.

Finally, a stepwise logistic regression was carried out to identify predictors of the intention to undergo breast cancer screening (Hosmer and Lemeshow 2000).

## Results

### Characteristics of participating women

The survey conducted among 1100 female patients demonstrated a remarkable response rate of 92% to the proposed questionnaire. The active engagement and participation of the medical community were highlighted, with 1014 patients providing comprehensive and informative responses. The age of the women ranged from 18 to 71 years, with a mean of 40.94 years (SD = 11.45 years). 77.5% were married, 11.7% were single, and 10.8% were widowed or divorced. Among the surveyed women, 50.9% were illiterate, and 21.8% had completed primary education. Regarding their occupation, only 18.5% were actively employed at the time of the survey, with 14.2% working in various occupations such as laborers, seamstresses, and domestic workers, etc.

### Knowledge of women regarding breast cancer

The results of this study showed that the average knowledge score was 67%. Out of a total of 1014 participants, 294 (29%) were classified as having "poor knowledge," while 720 (71%) were classified as having "good knowledge." Nearly, all the women surveyed (96.7%) reported being aware of breast cancer. However, when asked to provide a precise definition of the disease, only 34.9% were able to give appropriate answers (e.g., breast swelling or a lump), while others expressed incorrect or incomplete notions.

Regarding the sources of information on breast cancer, mass media, especially television, were the main channels of information for 92.1% of the women surveyed. In contrast, healthcare professionals were mentioned as a source of information by only 13.5% of the participants.

Concerning the risk factors of breast cancer, more than half of the women surveyed (51%) either did not know them or mentioned them inaccurately. Among those who could identify risk factors, unnatural and high-fat diet were most frequently mentioned (11.7%), followed by a family history of cancer (9.1%), and lack of breastfeeding (7.2%).

Regarding the means of preventing breast cancer, a natural and balanced diet was considered the main preventive measure by 31.5% of the women surveyed, followed by early cancer screening (21.2%), and regular doctor consultations (18.3%).

As for knowledge about breast cancer treatment, 34.9% of the participants stated that early detection and management of the disease were essential. Additionally, 33.5% mentioned tests, treatments, or medical consultations as means of treatment.

The use of conventional treatments such as chemotherapy was also mentioned by some participants, with 11.7% referring to chemotherapy alone and 3.3% mentioning the combination of chemotherapy with radiation therapy or surgery. However, it is concerning to note that 5.4% of the participants stated that they did not know which treatment options were available for breast cancer.

Regarding the different methods of breast cancer detection, the majority of participants (51.4%) stated that they did not know how breast cancer was detected. Among those who were aware of detection methods, medical consultations (check-up) were mentioned by 18.7%, while 15.4% mentioned ultrasound and/or mammography as screening methods.

However, it is alarming to find that only 2.7% of participants mentioned specific symptoms such as fatigue, breast pain, or the presence of a lump as methods of breast cancer detection.

### Determinants of women's knowledge regarding breast cancer

The determinants of women's knowledge regarding breast cancer are presented in Table 1. The results show that age, level of education, employment status, family type, number of children, place of birth, social and medical coverage, and regular medical consultation are all significantly associated with women's knowledge of breast cancer (*p* < 0.001).

**Table 1:** Determinants of women's knowledge regarding breast cancer

	Poor knowledge	Good knowledge	*p* value
	*N* (%)	*N* (%)	
Total	294 (29)	720 (71)	
Age			** < 0.001**
18–30	39(21.5)	142 (78.5)	
30–40	76 (24.1)	239 (75.9)	
More than 40	179 (34.6)	339(65.4)	
Marital status			0.087
Single	32 (27.1)	86 (72.9)	
Married	220 (28)	565 (73)	
Widower	31 (41.9)	43 (58.1)	
Divorced	11 (30.6)	25(69.4)	
Level of education			** < 0.001**
Illiterate	115 (22.4)	399 (77.6)	
Primary	105 (47.7)	115 (52.3)	
Secondary	58(36.2)	102(63.8)	
Superior	15(12.8)	102 (87.2)	
Professional activity			** < 0.001**
Yes	89 (43.2)	117 (56.8)	
No	205 (25.4)	603 (74.6)	
Type of family			** < 0.001**
Nuclear	142 (19.4)	589 (80.6)	
Extended	38 (44.1)	49 (55.9)	
Composite	74 (52.5)	67 (47.5)	
Number of children			** < 0.001**
00	48 (30.2)	111 (69.8)	
01	19 (15.2)	106 (84.8)	
02	63 (25.5)	184 (74.5)	
03	55 (23.2)	182 (76.8)	
04	46 (34.8)	86 (65.2)	
05 and more	63 (55.3)	51 (44.7)	
Place of birth			** < 0.001**
Urbain	122 (22.6)	419 (58.2)	
Periurbain	98 (48.5)	104 (51.5)	
Rural	74 (27.3)	197 (72.7)	
Social protection			** < 0.001**
Yes	130 (21)	490 (79)	
No	163 (29.1)	225 (70.9)	
Regular medical consultation			** < 0.001**
Yes	37 (9.4)	358 (90.6)	
No	256 (41.4)	362 (58.6)	

When a chi-square test result is associated with more than one degree of freedom, we used the calculation of the residuals (raw, standardised and adjusted residuals)

The bolded *p* value indicates a significant difference with a significance level below 0.05

Compared to younger women, those aged over 40 tended to have lower awareness of breast cancer. Similarly, women with higher education levels had higher knowledge than those with lower education levels (87% vs. 77%).

Regarding employment status, women who were employed generally reported having lower knowledge compared to unemployed women (56.8% vs. 74.6%).

Family type also appeared to play a role; nuclear families had higher knowledge than extended or composite families (80% vs. 55% and 47%).

Moreover, there was a significant negative correlation between the number of children and knowledge level. It was found that women with only one child had higher knowledge (84%), while women with five or more children had the lowest knowledge (44%).

Place of birth (urban, peri-urban, or rural) also had an impact on women's knowledge; women born in rural areas had more in-depth knowledge, with a rate of good knowledge of 72%, while only 58% of women born in urban areas had in-depth knowledge.

Furthermore, women who had social and medical coverage generally had more knowledge about breast cancer compared to those without coverage (79% vs. 70%).

Lastly, better knowledge of breast cancer was associated with regular medical consultation. Marital status was not found to be associated with the level of knowledge.

### Predictors of the intention to undergo breast cancer screening

Table 2 presents the results of binary logistic regression. We found that regular medical consultation, good knowledge about breast cancer, recommendation to undergo screening, professional activity, number of children, and level of education were predictive factors of breast cancer screening intention. Remarkably, regular medical consultation, good knowledge about breast cancer, and Recommendation to undergo screening were strong predictors of this intention. In fact, women who had regular consultations with doctors or health centers had an intention to undergo screening 15 times higher than women who did not consult (OR 15.295; 95% CI 6.369–36.731).

**Table 2:** Predictors of breast cancer screening intention

Variables	Coefficient	Odds-ratio (OR)	CI (95%)	*p* value
Regular medical consultation	2.728	15.295	6.369–36.731	0.000
Good knowledge about breast cancer	2.111	8.255	5.303–12.850	0.000
Recommendation to undergo screening	1.191	3.291	1.976–5.482	0.000
Professional activity	1.037	2.820	1.680–4.733	0.000
Number of children				0.007
0	0.361	1.434	0.660–3.117	0.363
1	1.174	3.235	1.285–8.143	0.013
2	0.723	2.062	0.998–4.257	0.051
3	1.077	2.937	1.397–6.175	0.004
4	0.033	1.033	0.483–2.211	0.933
Level of education				0.000
Illiterate	0.658	1.931	1.068–3.492	0.030
Primary	-0.421	0.656	0.348–1.238	0.193
Superior	1.011	2.748	1.208–6.251	0.016

Overall prediction percentage of the model: 87.8 Probability Chi-2 = < 0.001

R2 Nagelkerke: 0.63

Moreover, women with a good level of knowledge about breast cancer, its factors, diagnosis, symptoms, and screening had a tendency eight times higher to have an intention to undergo screening compared to women with low knowledge (less than 60%) (OR 8.255; 95% CI 5.303–12.850). Women who reported being advised to undergo screening by healthcare providers or family members were three times more likely to have a strong intention to undergo screening than women who did not receive any recommendation (OR 3.291; 95% CI 1.976–5.482).

Additionally, besides these three strong predictors, the intention to undergo screening was associated with professional inactivity (OR 2.820), having one to three children (OR between 2.062 and 3.235), and higher education level (OR 2.748).

## Discussion

Our study showed a moderate level of knowledge exceeding 60% among a total of 1014 participating women. These results are similar to those found in the UAE, where 65% of women demonstrated moderate knowledge. Additionally, 7.6% had good knowledge, and only 19% had poor knowledge (Abbas and Baig 2023). In contrast to a study in Syria that showed women had inadequate knowledge regarding breast cancer and its characteristics, such as risk factors, warning signals, and challenges of seeking medical care (Bohsas et al. 2023).

Our study also showed a higher level of knowledge compared to women in India. A study conducted with 480 women in India in 2020 revealed that only 49% of them were aware of breast cancer. However, Indian women mentioned several aspects to describe breast cancer more than the women in our study. For instance, 75% of Indian women mentioned nodules, 57% mentioned changes in breast shape and size, 56% mentioned nodules under the armpit, and 56% mentioned pain in a breast. Several risk factors were mentioned in India, such as early menstruation, late menopause, hormonal therapy, late pregnancy, and obesity (19%) (Prusty et al. 2020).

In Brazil, among the 417 female participants aged over 20 years, 203 had good knowledge about breast cancer risk factors and their prevention. Therefore, only 48% of women had good knowledge (Freitas and Weller 2019).

Indeed, our study showed high knowledge compared to other studies. However, on a qualitative level, Moroccan women were not able to describe the characteristics of breast cancer in detail.

Regarding the determinants of breast cancer knowledge, our study revealed that knowledge was associated with age, level of education, employment status, family type, number of children, place of birth, social and medical coverage, and regular medical consultation.

A study conducted in the UAE showed that women aged between 46 and 55, with a higher level of education, who received regular information from healthcare professionals, or attended awareness events, had a higher level of knowledge scores (Abbas and Baig 2023).

This parallels our study in some age categories, while a contrary observation was made in others. In the UAE, a positive association between age and knowledge was noted, whereas in Morocco, a negative association was observed. A similar contradiction was found in a study conducted in Saudi Arabia, where advanced age was linked to higher levels of knowledge. Additionally, other characteristics were associated with knowledge in that country, such as region of residence and education level, similar to our study. However, this particular study focused only on students (Shubayr et al. 2022).

Education level remains the strongest determinant and factor linked to women's knowledge. Thus, participants in a study in Syria with a higher education level (PhD) were more likely to have good knowledge regarding breast cancer, its symptoms, and risk factors compared to women with a primary education level (Bohsas et al. 2023).

The same results were found in India, where education level was the only variable significantly associated with awareness regarding breast cancer, showing a positive correlation. Indeed, women with 10 years of education were four times more likely to have a better awareness of breast cancer than other women (Prusty et al. 2020). In Brazil, in addition to education level, other socio-economic variables were associated with women's knowledge about breast cancer, such as income and employment status (Freitas and Weller 2019).

Regarding the prediction of the intention to undergo breast cancer screening, our study showed that regular medical consultation, good knowledge about breast cancer, and a recommendation to undergo screening were strong predictors of this intention, along with other variables such as level of education, professional activity, and number of children. Indeed, women's knowledge has been considered a predictor of this intention in the literature. A similar study to ours conducted in Korea with a total of 1609 women aged between 40 and 69 years found that women with a good level of knowledge about breast density were more likely to undergo breast cancer screening (Tran et al. 2021).

A survey conducted in five European countries showed results similar to ours among over 1000 women. Women who were informed about breast cancer screening and those who believed that their close relatives thought they should undergo breast cancer screening were more likely to participate in the screening. Additionally, a low level of perceived barriers to screening was a predictor of the intention to undergo screening (Ritchie et al. 2022).

The knowledge of women and the social norms of their close ones encouraging them to undergo screening were also strong predictors among 3000 rural women in China. Additionally, this Chinese study showed that personal factors, including distance, transportation, busyness, etc., and perceived behavioral control, as well as cultural-social factors, such as embarrassment from a physician, could also predict the intention of these women to undergo screening (Sun et al. 2022).

Contrary to our results, a recent study conducted in Spain using structural equation modeling to model the intention of undergoing screening concluded that there is no direct or indirect effect of knowledge on the intention to undergo screening. Instead, the influence of other behavioral variables on how people make decisions must be taken into account in the analyses. It was observed in this study that providing women with information about both the benefits and risks of screening does not affect their intention to undergo the test; however, certain personal characteristics such as concern about the disease or a long-term perspective do influence it. Although individuals' subjective characteristics are not easily modifiable, according to this study, the personalization of the screening program is necessary. A non-violent communication and shared decision-making between women and healthcare providers could allow women to discuss their preferences and fears with healthcare professionals (López-Panisello et al. 2023).

The results highlight the need to strengthen education about breast cancer, its characteristics, and screening. It is important to implement a program of education and regular consultations for women at healthcare centers. In this program, personalized breast cancer screening is recommended to improve women's knowledge, taking into account their individual characteristics, especially their level of education, which is a significant determinant of this knowledge. In Morocco, where the illiteracy rate is high, adapted communication by peers is essential to facilitate the acquisition of knowledge, especially for illiterate women or those with only primary education.

## Conclusion

Our study indeed showed a relatively high level of declarative knowledge among women concerning breast cancer (67%). However, the consistently low participation rates in breast cancer screening in Morocco indicate that the positive intention associated with elevated knowledge among Moroccan women is not always translated into actual practice. This calls for further in-depth research on the decision-making process, the barriers hindering the transition from intention to actual practice, and the potential for personalized education programs, especially for women with low levels of education or barriers to seeking regular medical consultations.

## Data Availability

The datasets generated during and analyzed during the current study are available from the corresponding author on reasonable request.
